# Researchers’ Needs for Resource Discovery and Collaboration Tools: A Qualitative Investigation of Translational Scientists

**DOI:** 10.2196/jmir.1905

**Published:** 2012-06-05

**Authors:** Suresh K Bhavnani, Michael Warden, Kai Zheng, Mary Hill, Brian D Athey

**Affiliations:** ^1^Institute for Translational SciencesUniversity of Texas Medical BranchGalveston, TXUnited States; ^2^Elsevier, Inc.Ann Arbor, MIUnited States; ^3^School of Public HealthDepartment of Health Management and PolicyUniversity of MichiganAnn Arbor, MIUnited States; ^4^School of InformationUniversity of MichiganAnn Arbor, MIUnited States; ^5^Medical School Information ServicesUniversity of Michigan Medical SchoolAnn Arbor, MIUnited States; ^6^Department of Computational Medicine and BioinformaticsUniversity of Michigan Medical SchoolUniversity of MichiganAnn Arbor, MIUnited States

**Keywords:** Translational research, collaborative computing, Clinical and Translational Science Awards, CTSA, Resource Discovery

## Abstract

**Background:**

A critical aspect of clinical and translational science (CTS) is interdisciplinary and collaborative research, which increasingly requires a wide range of computational and human resources. However, few studies have systematically analyzed such resource needs of CTS researchers.

**Objective:**

To improve our understanding of CTS researchers’ needs for computational and human resources in order to build useful and useable supporting informatics tools.

**Methods:**

We conducted semistructured interviews of 30 CTS researchers from the University of Michigan, followed by qualitative analysis of the interview transcripts.

**Results:**

The analysis identified three recurring themes: the need for the federation of information, the need to address information overload, and the need to humanize computing, including strong and well-informed views about the use of social networking tools for research collaboration. These findings helped us to narrow down the available design choices for assisting CTS researchers, and helped to identify potential deficiencies of well-known theoretical frameworks used to guide our study, with suggestions for future remedies.

**Conclusions:**

The user needs identified through the study, along with concrete design suggestions, provided key design, methodological, and theoretical insights, which are being used to guide the design and development of a CTS resource portal. The results and interview instrument should be useful to other institutions with Clinical and Translational Science Awards that face similar challenges related to helping CTS researchers make more effective use of computational and human resources.

## Introduction

Despite billions of dollars invested in biomedical research over the past five decades, there is a growing realization that our ability to generate medical breakthroughs far exceeds our ability to apply those results in improving clinical care and population health. The US National Institutes of Health responded to these challenges by advocating and funding a new approach known as clinical and translational science (CTS) [1]. The goal of CTS is to “accelerate the translation of laboratory discoveries into treatments for patients, to engage communities in clinical research efforts, and to train a new generation of clinical and translational researchers” [2].

The overall CTS approach is intended to encourage the 2-way translation of scientific discoveries between bench science, clinical research, and community-engagement activities, with the ultimate goal of improving human health. The growing acceptance of this approach has increased the motivation for researchers to work with collaborators across multiple disciplines. This shift has resulted in the need to use a wider range of computational and human resources. For example, basic scientists who wish to translate results from the laboratory to the design of clinical trials need access to computational resources within and across disciplines such as gene expression analysis and patient registries, and to identify and work with collaborators such as data mining experts and clinicians.

In recognition of these needs, several recipients of the Clinical and Translational Science Awards (CTSA) such as Harvard University and Vanderbilt University have already invested substantial effort in building Web portals to facilitate the discovery and use of computational and human resources. Furthermore, almost US $30 million in federal funding has been allocated to CTSA sites for creating tools that enable researchers within and across institutions to communicate, collaborate, and discover resources more efficiently and effectively [3]. Other recipients of CTSA have partnered with commercial social networking platforms to create new resources for CTS researchers [4]. However, while some publications describing these efforts have targeted specific aspects of building research networking systems such as their interoperability [5], few studies have systematically analyzed the resource needs of CTS researchers to inform the design of such systems. For example, a recent set of studies (eg, [6-8]) conducted at the University of Pittsburgh attempted to identify the general services and functions needed specifically for locating experts to facilitate collaboration, leading to the design of a system called Digital|Vita. However, to the best of our knowledge, there appears to be no published study that has directly probed the broad computational and human resource needs of CTS researchers and engaged them in designing an informatics solution.

We were therefore motivated to conduct a systematic investigation to directly engage CTS researchers in articulating their resource needs and to help guide the design of future solutions. Systematically understanding users’ needs and engaging them in the design process is important for two reasons. First, users can help narrow down the range of possible solutions and avoid arbitrary choices purely based on expert opinions rather than on facts and evidence. For example, many CTSA institutions are exploring whether to build social networking tools for researchers but lack guidelines to help make such a decision or to identify key functionalities to implement. Second, empirical studies have repeatedly shown that computerized systems have been poorly adopted and underused in health care institutions, which is often due to the mismatch between delivered designs and the preferences and constraints of the intended user population [9]. Such low adoption rates are underscored by technology acceptance models (TAMs), which have shown that the user acceptance of a technology is strongly influenced by the perceived usefulness and perceived ease of using that technology (eg, [10]). Furthermore, prior research has shown that involving potential users early in the development process of systems can help increase ownership of the final product and hence the likelihood of adoption [11].

In this paper, we describe the results of a study in which we engaged junior and senior CTS researchers at the University of Michigan in a qualitative study. Our goal was to identify the computational and human resource needs of the researchers, elicit concrete suggestions for the design of future systems, and engage the researchers in an iterative participatory design process.

## Methods

We conducted our study at the University of Michigan (a CTSA site), which has 7600 faculty members, of whom 750 have been served by the CSTA and therefore can be considered CTS researchers. The University is also home to information technology departments that develop and customize information technology solutions across the medical campus. The leadership at the University of Michigan strongly recommends faculty and students to engage in interdisciplinary collaborations. Furthermore, since its establishment, the CTSA at the University of Michigan has received an increasing number of requests for accessing a wide range of human and computational resources. Given this need, and because the resource needs of CTS researchers had never been systematically analyzed, the CTSA leadership strongly supported conducting such a study. We therefore designed a semistructured interview study inviting a wide range of CTS researchers to help address the research question: *What are the computational and human resource needs of CTS researchers, and how can we translate them into designing useful and useable informatics solutions supporting interdisciplinary CTS research?*

The goal of the semistructured interviews was to enable an understanding of the motivations for the resource needs at a conceptual level, which would then guide the design of a survey with the goal to elicit information about specific resources (eg, project management applications) required by the researchers. We were also motivated to build a participatory design [12] relationship with the researchers so that they could be involved in the iterative refinement of future informatics solutions.

The design of our study was guided by two existing theoretical frameworks. First was the TAM and its latest extension, referred to as the unified theory of acceptance and use of technology. This theory postulates that performance expectancy (perceived usefulness), effort expectancy (perceived ease of use), social influence, and facilitating conditions are four important factors underlying end users’ decision to accept or reject a technology, in addition to other moderating factors such as age and experience or seniority [10,13,14]. Some researchers (eg, Venkatesh et al) argue that this family of models accounts for a considerable proportion of behavioral intention variance in explaining individuals’ technology acceptance and usage decisions [13]. Second was technology-mediated collaboration research [15-17], whose results include (1) the importance of face-to-face collaborations for building trust, and (2) the importance of designing new collaboration technologies that are compatible with existing norms and practices so as to increase the probability of adoption.

### Study Design

We used semistructured interviews to collect research data. Semistructured interviews are a well-known qualitative method [18] used in a wide range of fields including human–computer interaction, sociology, and medical informatics. The method is most useful when topics of research interest have been identified, but there is a lack of understanding of those topics about which to ask structured questions such as in a survey. The goal of the semistructured interview method is, on one hand, to focus an interview based on an ordered list of predetermined questions and, on the other hand, to enable the interviewer to explore issues that emerge during the interview, often leading to unexpected insights. This is achieved by asking open-ended, predetermined questions to enable users to discuss a topic. Depending on the answers, open-ended questions are then often followed opportunistically by carefully worded nonleading prompts to encourage continued elaboration, and probes to explore emergent issues or to guide the discussion in promising directions. Such interviews typically amass a large amount of in-depth qualitative data, and the studies are generally conducted with tens rather than hundreds of participants.

The two theoretical frameworks discussed above helped to guide the overall design of the study, in addition to the semistructured interview instrument. The theories on individual technology acceptance motivated us to include both junior and senior researchers in our study, as they might possess different perspectives regarding CTS research and therefore distinct needs for resources. In addition, the theories prompted us to include in the interview protocol key questions soliciting important theoretical constructs, such as the researchers’ perceived barriers to using the proposed informatics tools for conducting interdisciplinary translational research, and the social contexts in which they are situated that may convey salient social cues influencing their own beliefs of, and attitudes toward, such tools. Furthermore, the research on technology-mediated collaboration motivated us to ask three broad questions that were specifically related to the research process: how the researchers conducted or supported research activities, the nature of their scientific collaborations, and the tools that they used to conduct such research.

By using guidelines for interview design [19], we operationalized the above theoretical constructs into an interview instrument that proceeded from demographic and background questions to descriptive questions and broader opinion questions, and ended with user appraisals of two hypothetical designs: a Web portal concept integrating scattered information sources, and a digital curriculum vitae system that aggregates information about faculty and facilitates expertise finding. The resulting instrument consisted of 10 questions divided into 4 sections: (1) introduction and consent, (2) background and role, (3) research process, and (4) suggestions for supporting translational research. The interview protocol is provided in Multimedia Appendix 1.

To recruit the CTS researchers, we used a 2-step snowball sampling method [20]. First, we requested 15 researchers who held leadership positions in the CTSA at the University of Michigan to participate in the study. Then, we requested the above researchers to identify junior faculty whose work was closely related to CTS. Among the junior faculty identified, we selectively invited a subset to participate in the study in order to ensure the representativeness of major disciplines and academic departments. The overall recruitment method resulted in 15 senior and 15 junior CTS researchers. These researchers were affiliated with a broad range of schools (eg, medicine, public health, nursing, and pharmacy) and had different backgrounds (eg, basic science, clinical and health services research, and community-based research), and therefore represent the breadth of CTS as it is currently conceptualized. Table 1 summarizes the characteristics of the study participants.

**Table 1:** Participant characteristics (N = 30).

Characteristic		Total number of participants	Junior investigators	
			N	%
**School**				
	Medical School/Health Systems	23	12	52%
	School of Public Health	4	2	50%
	School of Nursing	2	1	50%
	College of Pharmacy	1	0	0%
**Gender**				
	Female	12	9	75%
	Male	18	6	33%

Each of the interviews was conducted by an information technology business analyst (MW) and a biomedical informatics faculty member (SKB or KZ). All interviews were conducted in the office of the researcher. Each interview lasted approximately 90 minutes and was digitally recorded in audio format. The University of Michigan Institutional Review Board reviewed and approved the research protocol.

### Data Analysis

The 30 audio-recorded interviews were transcribed by a professional transcriber resulting in approximately 600 pages of text. The data were subsequently analyzed in 3 steps. First, two analysts (MW and KZ) used the technique of open coding and categorization to iteratively annotate sections of the transcripts. This was achieved by using the constant comparison method [21] facilitated by the NVivo qualitative data analysis software (QSR International, Doncaster, Victoria, Australia). Second, a third analyst (SKB) independently analyzed and refined the thematic coding results. Third, all three analysts used affinity diagrams to reach a consensus on the final categorization of the data and identified implications of the results for a concrete design proposal.

## Results

Analysis of the qualitative data helped to identify three emergent interrelated themes (and their subthemes), which captured the computational and human resource needs of CTS researchers. We first present evidence for these themes, followed by an analysis of the nature and causes of sentiments underlying those needs.

### Emergent Themes Related to Resource Needs

As Table 2 shows, analysis of the interviews helped to identify three interrelated themes: (1) the need for the federation of information, (2) the need to address information overload, and (3) the need to humanize computing. In the following sections, we discuss each of these categories with examples of verbatim quotes provided by the study participants.

**Table 2:** Themes of user needs and design suggestions identified through the qualitative analysis of the interview transcripts.

Theme	Subtheme		Description
**1. Need for the federation of information**			
	Discoverability	Difficulty in discovering new information and resources	
	Structured vs scattered information presentation	Disorientation caused by scattered information and resources	
	*Design: *centralized access to resources	Desire for a centralized, authoritative location for information and resources	
**2. Need to address information overload**			
	Relevant vs irrelevant information	Concern that informatics solutions will cause more information overload	
	Push vs pull of information	Concern about how to balance the push and pull of information	
	*Design: *comprehensible and personalized information	Desire for information presentation that is comprehensible and filtered based on personal preferences	
**3. Need for humanized computing**			
	Human vs computer aided	Negative perceptions of tools that assume that research expertise can be found without humans	
	User control vs automatic control	Negative perceptions of tools that will result in loss of control	
	*Design: *human in the loop	Desire for human-aided services (eg, “concierge services” and “online consultation”) to facilitate finding research expertise	

#### The Need for the Federation of Information

A common theme in the data was the need for the federation of information in three overlapping subthemes. First, there was an awareness of the large number of scattered resources across the clinical, research, education, and administrative systems. For example:

there are so many different sites and so many different tools—like you don’t know where to start

Next, several stated that, while new tools are beneficial to their research, they had difficulty *discovering *that they existed.

Our problem now is we’ve got [new resources and research expertise] and some of the people don’t even know we have [them]...somewhat the hardest part is figuring out what resources are out there, and who is out there

The above general awareness of useful but scattered and difficult-to-discover resources resulted in a strong desire for a central location where the resources could be accessed.

...the more things you can bring under one portal would be fantastic, and that sounds like something that would be very useful if it’s customizable

The results therefore confirmed that CTS researchers were indeed attempting to use a wide range of resources, but that those resources were highly scattered and it was difficult to know that they existed.

#### The Need to Address Information Overload

The researchers expressed concerns that any solution for integrating scattered resources needed to be designed carefully so that it would not further exacerbate the issue of information overload. They expressed this need in two ways. First, they expressed the need that system designers should develop systems that help to quickly distinguish between relevant and irrelevant information.

I know your business is really more of managing the data and mining it and stuff, but I mean people that are in the interface business, I mean we’re in information overload...how do you distinguish between important information and nonimportant information? Because no one wants to waste their time.

In the absence of such tools, they had developed strategies to cope with information overload, such as using manual collaborative filtering techniques.

I skimmed through my emails quickly and deleted most of the event announcements without reading in full detail...I know that if something is really important to me, it will come back again later, for instance referred to me by my colleagues

Second, they expressed concerns about how to balance the push and pull of information. On one hand, the researchers were concerned that an excessive amount of information of little relevance will be pushed to them. On the other hand, if information is not pushed to everybody, the collaborative filtering strategy may no longer work.

You need like an optimal level of notification. If it’s too much, people will disregard it. If it’s not enough, you know...I don’t think I’ll look at another website just to see if potentially there’s some collaborator.

The researchers provided several recommendations to address the information overload problem. These included interfaces that structured information for easy comprehension:

...so much information overload that you don’t see the forest through the trees...[Design] something that doesn’t have so much there that you don’t in a sense know where to begin

Another suggestion was to build a system that provides tailored content based on each user’s research area:

I am afraid I will not have time to customize it extensively, so I would much prefer if the tool can use some intelligence to predict my preferences based on my research areas.

#### The Need for Humanized Computing

The researchers expressed strong concerns about future tools that would dehumanize interpersonal relationships that are crucial for successful research collaborations. For example, they expressed suspicion toward the usefulness of the concept of automated research-expertise finding:

I realize there’s a lot of informatics enthusiasm for collaboration ware for things like basically medical and professional versions of Facebook...That is beloved of informaticians, but frankly I don’t think it’s really going to fire up a lot of other people

In particular, there was widespread concern among junior as well as senior researchers that social networking applications would not be able to convey information about potential collaborators, such as whether a researcher is trustworthy. Such information was often implicit in personal referrals or direct communications. For example, a junior researcher stated

[Such tools] can find expertise; however, what it lacks is “personal touch”...if my boss says you should talk to [name of a researcher]...like, he knows this person and he knows me...there is this personal touch because he is vouching for this person and there is some element of trust

Some of the senior researchers were concerned that being automatically enlisted in social networking tools would result in a large number of requests for new collaborations that they did not need. Such reservations, as one researcher noted, lead to the paradox of using social networking tools for research:

...the paradox is the types of people that you most want to have their information up-to-date because they might be able to be the most helpful to other people are the ones that are least like[ly] to do it...the junior faculty that have a lot of time will make sure that everything about themselves is amazingly accurate, but how many people are going to need to find a junior faculty member...?

The researchers provided several concrete recommendations for designing future systems that could overcome the concerns of dehumanized systems. These included systems that incorporated the human in the loop for finding resources:

...you basically say [on the website] “Did you get what you need, and if not type [it] in. If you could wait a couple of hours someone will respond by email,” but at the bottom it’s like “Don’t leave this page unless you got what you wanted.”

Another recommendation was for displaying social information of resource usage such as which tools are used by whom, with the goal of helping discover new resources based on what others are using.

### Sentiments Underlying Resource Needs

While the above emergent themes directly addressed our goal of identifying the overall resource needs of CTS researchers, we were unprepared for the strength of the sentiments expressed during the interviews. During the interviews, the researchers required almost no prompting to discuss their needs, and most interviews ran over the 1.5 hours allotted, with 2 researchers inviting us back for follow-up interviews. We therefore probed deeper into the interview transcripts, in addition to reflecting on our personal face-to-face encounters with the researchers, to uncover cross-cutting themes that could explain the strong sentiments expressed during the discussions on resource needs.

Our analysis revealed that the strength of the sentiments did not appear to arise from an abstract conception or ignorance of new technologies, but rather were grounded in concrete interactions with university-based and contemporary Web-based technologies. For example, when discussing the need for a portal that federated resources, a researcher reflected on his use of a university-based system for institutional review board reviews:

When I do my reviews for the MCRU [Michigan Clinical Research Unit], the GCRC [General Clinical Research Center] legacy, I try to do them directly on eResearch. Some of my colleagues just threw up their hands and said: “Just send me a paper copy because I just can’t do it. It’s so inefficient that I don’t even want to learn. It’s so arcane and so unwieldy I just can’t even deal with it. So just send me a paper copy or I’ll have my administrative assistant print off a paper copy, and I’ll mark up the copy and send it back to you because I can’t deal with it.”

In the same vein, another researcher complained about the large overheads related to the CTSA-wide interactions:

This thing has grown into some kind of enormous effort that we didn’t anticipate. It’s kind of sad when you look at these proposals...oh, we will participate in the national level, blah, blah, and you say “Be careful what you wish for, guy”...it’s an unfunded mandate basically...It’s out of control. I think there’s too much. There’s too many wikis, too many things, too much need to interact with others, and this without it being paid for, and knowing this is a real problem.

In addition to experiences with university-based systems, the researchers were not naïve about contemporary Web-based systems, with equally strong sentiments. For example, a researcher, when discussing his use of browser Favorites, stated:

You read journal articles and you decide there’s something new, and you might visit [it]. It references a website, and you might visit the website and decide “Well that’s pretty good—I’m going to put it on my Favorites list.” So I might put it on a Favorites list and say “Do I really go [to the Favorites list]? Do I gain anything by going there?”...I know I could do a Google search, and get [to the saved website] if I needed to.

Another researcher described the tensions involved in maintaining the accuracy of information in systems designed for enabling collaboration:

I mean there’s a bit of a paradox here in that as you get busier, you’re less likely to do these things and to check whether the information is accurate or whatever. There’s so many different things that get sent to me about the U of M alumni club and stuff like that. I have never gone and looked to see if it’s accurate. If it’s accurate, it’s accurate; [if] it’s not, then people won’t get a hold of me...So I think that we just have to be mindful of this as we construct any kind of [system].

However, the strongest sentiments pertained to the use of social networking tools (eg, Facebook) for research collaboration:

Teens are really cool on Facebook because they’re big into the social networking aspects of it, but it’s the social interaction that draws them there. They’re there because their friends are there. If you’re trying to build an online community where people who don’t even know each other yet, you don’t have that draw. It’s always going to be a little in-club that’s into that kind of thing. You’re not going to get the people you’re looking for. If you want the people you’re looking for, face time, there’s no substitute. Facebook is not a substitute for face time. You’ve got to get people together in a room who wouldn’t normally be together to hear about what they’re talking about.

Besides being well informed, this sentiment about social networking did not seem related to a generational divide, based on our interview of a junior researcher aged 27:

I have a Facebook account but I never check it—just if people put photos on it—I use it to go look up the photos. But I’m not into that very much.

The sentiments surrounding resource needs therefore appeared to be grounded in real-world experience and knowledge of contemporary systems, a realization that strongly influenced our conceptions of how to design future solutions.

## Discussion

Most biomedical applications are built without an adequate consideration of user needs and design involvement, which has been a major impediment to system adoption and long-term acceptance [9,22]. In most cases, users are asked for opinions after the systems are built or purchased, which strongly biases the developers toward asking questions and making convenient modifications that protect investments and prior design decisions, rather than serving the real needs of the users. When deployed systems fail to be adopted, it is often too late and difficult to make the significant changes needed for success, resulting in wasted time, effort, and resources [9,23]. This point is critical, as large amounts of funds are being spent to develop systems with perceived uses, rather than uses that are grounded in the needs of actual users.

Our approach was aimed to avoid such pitfalls by (1) probing the needs of users *before *system design and development began, (2) using the semistructured interview method, which allowed us to ask targeted questions while at the same time enabling users to express unexpected views that directly confronted our hypotheses and biases, (3) soliciting opinions for potential solutions, and (4) increasing the perception of ownership among a few key potential users, who could become champions to promote the informatics tools eventually developed.

Our main goals were to quickly narrow down the possible choices of system designs that would be useful and usable for CTS researchers, and to increase the chances for quick, widespread, and sustained adoption of the resulting system. To the best of our knowledge, this is the first study to systematically identify the resource needs of CTS researchers, with a focus on eliciting concrete design suggestions. Furthermore, our study was informed by the well-established and extensively validated TAM framework, in addition to extensive research in technology-mediated collaboration. Both frameworks guided us in the design of our study, enabling us to ask relevant questions during the semistructured interviews, which quickly yielded rich participant accounts that were critical to our research question. Furthermore, the 2-step snowball sampling method for selecting researchers from a wide range of backgrounds quickly enabled us to achieve those goals.

The resulting targeted study revealed that the researchers were frustrated about not being able to easily discover computational and human resources that were potentially useful, but were often hidden and scattered. However, the enthusiasm for a centralized location for such resources was tempered by cautions about developing solutions that increased information overload and dehumanized computing.

While the above results might not appear novel in themselves, they helped us make important decisions about how to proceed with our developmental efforts. However, we were unprepared for the strength of the sentiments expressed during our discussions of resource needs. These sentiments did not appear to be naïve responses to technological solutions, but rather were based on real-world experience of university-based and contemporary Web-based tools. In particular, we were struck by the strong sentiments specifically against borrowing the generic *social networking *tools (eg, Facebook) for the purposes of research collaboration without a thoughtful redesign. Based on several debates within the design team, this finding has forced us to reexamine how current social networking tools (used essentially to maintain weak ties [24] and therefore requiring a low degree of trust) could evolve into a new class of research networking tools (used for finding and sustaining research collaboration, and therefore requiring a high degree of trust). After all, it is well known that current social networking tools are rarely used to find and establish new contacts [24]. Research networking tools should, therefore, be carefully designed to address the complex combination of trust and cultural issues required for scientific collaboration before they can be expected to be widely adopted for finding and establishing new research collaborations. Furthermore, we found senior researchers who possess the expertise and resources desired by junior faculty members to be least enthusiastic about using and contributing to such tools. Their lack of motivation to contribute to a research network could severely affect the network externality of such tools, hence diminishing their value.

The overall results have several similarities and differences with the studies conducted at the University of Pittsburgh [6-8]. Methodologically, although both of our approaches had the similar goal of engaging end users to determine their needs and to guide design, the Pittsburgh studies were different in that they (1) were motivated in part by the low adoption of an existing custom university system designed for helping faculty to establish collaborations [7], (2) were focused specifically on tools for research collaboration [8], and (3) recorded the content of the 27 interviews of researchers through handwritten notes after each interview was completed [6,7]. In contrast, our interviews did not focus on specifically understanding the use and design of collaboration tools, but rather were focused on situating our understanding of computational and human resource needs in the broader context of research work practices of translational scientists. Furthermore, the digital recordings provided verbatim accounts, which enabled us to recall, detect, analyze, and present the nuanced sentiments underlying the emergent themes. However, despite these methodological differences, there were several similarities in the results. Both sets of studies revealed that researchers often used existing real-world connections to determine new collaborations and had low motivation for updating their public online profiles. In fact, Schleyer and colleagues acknowledge that senior, more well-established researchers “are so well-informed and well-connected that they, on average, will outperform any electronic system” [6]. They conclude by noting that research networking tools might therefore be more useful for junior researchers, contingent on an overall critical mass of researchers adopting the system. Future research should therefore investigate under which conditions junior researchers will use research networking tools, especially given the widespread availability and low cost of using powerful online tools such as Google, combined with the strong interpersonal advantages of using their mentors’ well-established collaboration networks.

The above findings have motivated us to pay careful attention to the sociotechnical issues of the researchers’ social status in the organization, and to provide appropriate incentive structures for collaboration and contribution when designing future solutions. As discussed below, these overall results have direct design, methodological, and theoretical implications.

### Design Implications

The three emergent themes, and our subsequent understanding of the nature and causes of the strong sentiments underlying them, helped us to narrow down the available design choices for assisting CTS researchers. Because of the urgent need to federate scattered resources at the University of Michigan, we prioritized our efforts to first target that goal by building a CTS resource portal, keeping in mind that it could be scaled up to include future resources such as research networking. Accordingly, our design provides functionality that includes (1) a scalable system that federates access to a wide range of resources, (2) a dashboard view (possibly using the *portlet *technology [25]) of current projects and other information that is initially customized based on the researchers’ backgrounds, (3) the ability to modify the displayed resources through easy customization, (4) the ability to discover resources that are ranked based on a user’s profile and what other researchers are using, and (5) a concierge service to guide researchers to humans if they are unable to find specific computational or human resources. These functionalities directly address the emergent themes in our study.

To enable the researchers to continue to be engaged in the design process, a prototype of the above system was implemented and presented to a subset of the researchers for feedback and design input. Our preliminary analysis suggests that the researchers have responded positively to the prototype. Our future research includes development of a survey to solicit similar feedback about the prototype from a larger number of CTS researchers beyond those we originally interviewed, and iterative refinement of the system based on the continued participation of the intended users.

### Theoretical and Methodological Implications

While our research focus was to precipitate a participatory design process, where we work closely with the researchers on progressive refinements of our informatics solutions, neither the technology acceptance theories nor the research on technology-mediated collaboration prepared us for the strong sentiments during our discussions on resource needs. These sentiments appeared to be directly related to past experiences with identical or similar technologies. While past experience could be theoretically interpreted as a behavioral antecedent to existing constructs of the TAM (eg, to performance and effort expectancy), it manifested so distinctively in our study that we believe past experience deserves closer attention in its own right, both theoretically and practically. Therefore, we believe our results suggest that, in future research, additional theories that have explicitly modeled past experience, such as those developed by researchers in marketing science, could be incorporated into studies of the design and acceptance of information systems [26-29]. Accordingly, we recommend that designers of future systems for CTS researchers (a unique user population with the specific goal of translational research) pay close attention to their end users’ past experiences with identical or similar technologies so that such experiences will be directly addressed in the design and presentation of new systems, with the goal of improving the chances of successful adoption and sustained use of the informatics solutions.

From a methodological perspective, it is also pertinent to note that over the course of our study and its presentations to various stakeholders, we have encountered strongly polarized views for the results. On one hand, translational researchers outside our study pool strongly identify with the results and often have their own contextualized accounts of similar sociotechnological issues. We have anecdotally encountered at other CTS sites almost identical issues related to computational resources that are not consolidated, difficult to use, and designed with strong assumptions of user needs, resulting in frustrations among researchers. On the other hand, technology developers and administrators, who are often suspicious of empirical studies, strongly criticize the results, using the argument that users are known to have difficulty articulating what they need and often base their needs narrowly on existing systems and biased views of new technologies. Instead, developers and administrators often propose a strategy to “build the system and users will come.” Unfortunately, such a strategy has not had a very good track record of sustained success, especially in the biomedical domain [9], often resulting in mandated use of poorly designed systems, coupled with contrived statistics for their success.

As professionals trained in the study of human–computer interaction and sociotechnical issues, we are acutely aware of such issues and have therefore espoused a strategy of participatory design [12], which early in the design process establishes a process of iterative refinement of solutions in partnership with users who, as the data show, are often impressively knowledgeable of complex sociotechnical concepts related to contemporary technologies. Our hope is that this methodological approach, besides reducing costly trial-and-error cycles, has a better chance of establishing long-term trust between researchers, administrators, and developers, leading to the sustained development and adoption of novel, useful, and usable informatics solutions for translational science.

### Conclusions

We believe that our study makes four contributions. First, it provides direct and detailed evidence of the resource needs of CTS researchers. Second, it identifies concrete recommendations and cautions for the design of informatics solutions to help find and use computational and human resources. Third, it provides a semistructured interview instrument and an example of how it can be used to guide the design of contextually relevant informatics solutions. Fourth, it identifies potential deficiencies of well-known theoretical frameworks used to guide our study and suggestions for future remedies. 

Our goal is to obtain feedback on our prototype using an iterative participatory design approach. Furthermore, we hope to conduct a survey to elicit the specific resource needs that should be included in the portal. The interview instrument and overall approach could therefore be used by other CTSA institutions to design current and future informatics solutions that are useful, usable, and contextually relevant to the populations that they serve, with the ultimate goal of accelerating progress in translational science.

## Multimedia Appendix 1

Semistructured interview instrument.

## Abbreviations

CTSA: Clinical and Translational Science Awards
CTS: clinical and translational science
TAM: technology acceptance model
