# Supplementary material for: Researchers’ Needs for Resource Discovery and Collaboration Tools: A Qualitative Investigation of Translational Scientists
Source: J Med Internet Res. 2012 Jun 5;14(3):e75. doi: 10.2196/jmir.1905 (PMC3415064; doi:10.2196/jmir.1905)
Supplement: Supplementary file 1 [file jmir_v14i3e75_app1.pdf]

Appendix A: Semi-Structured Interview Instrument

1. Introduction and Consent

A critical aspect of translational research is to enable the fluid transfer of information between different researchers. To enable this transfer of information, we wish to understand your needs for conducting effective translational research. All the data collected during this interview will be anonymized. We will greatly appreciate a recording of this interview so we can analyze it at a later time, and your consent for conducting and publishing the anonymized results of this interview.

2. Background and Role

Question-1: Can you please describe your current role in the university?

Possible Probes:

- Approximately how much time do you spend in research, teaching, and administrative duties?
- When did you come to the University of Michigan?

Question-2: What is the specific role that you play in the CTSA?

Possible Probes:

- How did you first get involved with the CTSA?
- What has been your experience with CTSA?
- What connection is there between your role in the CTSA and your role in the University?

Question-3: How do you see your role changing in the University and in the CTSA in the next five years?

Possible Probes:

- Do you anticipate a change in your organization, or in your University role that may affect your CTSA role? What may trigger that?

3. Research Process

Question-4: We are very interested in learning about the process of how you **conduct research** (for administrators this will be **support research activities**). Can you please describe a specific research project that you have worked (or are working) on, and how you addressed the research problem?

Possible Probes:

- What were the research questions (research administrative tasks)?
- In what way were the phases different?
- In hind sight, how would you do things differently? What consequent processes and outcomes would be different?
- Can you describe your experiences with collaboration leading up to your current role?

Question-5: Can you please describe if and how you collaborated with others to complete your tasks?

Possible Probes:

- (If there is little or no collaboration) What keeps you from collaborating more? Would you like to collaborate more with other researchers?
- (Else) Who are your collaborators (co-workers)?
- How did you find your collaborators?
- How often do you exchange information with your collaborators?
- How do you exchange information with collaborators?

Question-6: Can you please describe the kinds of tools you used in the project you described? (Please include specific examples.)

Possible Probes:

- Which tools do you use at home and at work?
- When do you use tools to conduct research?

4. Suggestions for Supporting Translational Research

Queston-7: Can you please describe your perception of translational research initiatives currently being conducted at the University of Michigan, such as in the CTSA?

Possible Probes:

- How do you get information about other CTSA-related research activities?
- What kinds of information do you get about other CTSA-related activities?
- What is the frequency at which you get information about other CTSA-related research activities?

Queston-8: Can you please describe where you would like to see translational research in your area of specialty at the University, and nationally?

Possible Probes:

- What are your major hurdles for conducting translational research?

Question-9: Can you please provide suggestions of how to improve our chances of achieving the goals of translational research at the University of Michigan, and across CTSA sites?

Possible Probes:

- What kinds of tools do you wish you had?
- What kinds of human support do you wish had?

Question-10: There has been a discussion on building a portal to integrate many resources, and a system to automatically construct an automatic Digital CV enabling expertise finding. Can you please tell us your views about such systems?

Possible Probes:

- Imagine you come into your office and start to use such a portal. What functionality would you need?

What interface do you see this system having?
